# Safety and Immunogenicity of an mRNA-1273 Booster in Children

**DOI:** 10.1093/cid/ciae420

**Published:** 2024-08-19

**Authors:** Vladimir Berthaud, C Buddy Creech, Christina A Rostad, Quito Carr, Liberation de Leon, Monika Dietrich, Anil Gupta, David Javita, Sharon Nachman, Swetha Pinninti, Mobeen Rathore, Carina A Rodriguez, Katherine Luzuriaga, William Towner, Anne Yeakey, Mollie Brown, Xiaoping Zhao, Weiping Deng, Wenqin Xu, Honghong Zhou, Bethany Girard, Roxanne Kelly, Karen Slobod, Evan J Anderson, Rituparna Das, Jacqueline Miller, Sabine Schnyder Ghamloush

**Affiliations:** Meharry Medical College—Division of Infectious Diseases, Clinical and Translational Research Center, Nashville, Tennessee, USA; Vanderbilt Vaccine Research Program, Vanderbilt University Medical Center, Nashville, Tennessee, USA; Department of Medicine, Emory University School of Medicine and Children's Healthcare of Atlanta, Atlanta, Georgia, USA; MedPharmics, LLC—Albuquerque, Albuquerque, New Mexico, USA; Center for Clinical Trials, LLC, Paramount, California, USA; Department of Pediatric Infectious Disease, Tulane University School of Medicine, New Orleans, Louisiana, USA; Dr. Anil K. Gupta Medicine Professional Corporation, Toronto, Ontario, Canada; Prohealth Research Center, Doral, Florida, USA; Renaissance School of Medicine, SUNY Stony Brook, Stony Brook, New York, USA; Division of Pediatric Infectious Diseases, University of Alabama at Birmingham/Children's of Alabama, Birmingham, Alabama, USA; University of Florida Center for HIV/AIDS Research, Education and Service (UF CARES), Jacksonville, Florida, USA; Division of Pediatric Infectious Diseases, University of South Florida, Morsani College of Medicine, Tampa, Florida, USA; Program in Molecular Medicine, UMass Chan Medical School, Worcester, Massachusetts, USA; Department of Infectious Diseases, Kaiser Permanente Los Angeles Medical Center, Los Angeles, California, USA; BioPoint Contracting, Wake Forest, North Carolina, USA; Moderna, Inc, Cambridge, Massachusetts, USA; Moderna, Inc, Cambridge, Massachusetts, USA; Moderna, Inc, Cambridge, Massachusetts, USA; Moderna, Inc, Cambridge, Massachusetts, USA; Moderna, Inc, Cambridge, Massachusetts, USA; Moderna, Inc, Cambridge, Massachusetts, USA; Moderna, Inc, Cambridge, Massachusetts, USA; Cambridge ID & Immunology Consulting, LLC, Somerville, Massachusetts, USA; Department of Medicine, Emory University School of Medicine and Children's Healthcare of Atlanta, Atlanta, Georgia, USA; Moderna, Inc, Cambridge, Massachusetts, USA; Moderna, Inc, Cambridge, Massachusetts, USA; Moderna, Inc, Cambridge, Massachusetts, USA

**Keywords:** mRNA-1273, COVID-19, booster dose, children, SARS-CoV-2

## Abstract

**Background:**

A 2-dose mRNA-1273 primary series in children aged 6 months–5 years (25 µg) and 6–11 years (50 µg) had an acceptable safety profile and was immunogenic in the phase 2/3 KidCOVE study. We present data from KidCOVE participants who received an mRNA-1273 booster dose.

**Methods:**

An mRNA-1273 booster dose (10 µg for children aged 6 months–5 years; 25 µg for children aged 6–11 years; age groups based on participant age at enrollment) was administered ≥6 months after primary series completion. The primary safety objective was the safety and reactogenicity of an mRNA-1273 booster dose. The primary immunogenicity objective was to infer efficacy of an mRNA-1273 booster dose by establishing noninferiority of neutralizing antibody (nAb) responses after a booster in children versus nAb responses observed after the mRNA-1273 primary series in young adults (18–25 years) from the pivotal efficacy study. Data were collected from March 2022 to June 2023.

**Results:**

Overall, 153 (6 months–5 years) and 2519 (6–11 years) participants received an mRNA-1273 booster dose (median age at receipt of booster: 2 and 10 years, respectively). The booster dose safety profile was generally consistent with that of the primary series in children; no new safety concerns were identified. An mRNA-1273 booster dose elicited robust nAb responses against ancestral SARS-CoV-2 among children and met prespecified noninferiority success criteria versus responses observed after the primary series in young adults.

**Conclusions:**

Safety and immunogenicity data support administration of an mRNA-1273 booster dose in children aged 6 months to 11 years.

**Clinical Trials Registration:**

NCT04796896 (Clinicaltrials.gov).

## Teaser

A booster dose of mRNA-1273 in children aged 6 months to 11 years was well tolerated and elicited robust immune responses against SARS-CoV-2, supporting recommendations for COVID-19 vaccination in individuals aged 6 months and older.

Coronavirus disease 2019 (COVID-19) is an important cause of severe respiratory disease among children, with associated hospitalization rates comparable to influenza [1, 2]. Most US children hospitalized for COVID-19 in early 2023 were unvaccinated or had not received an updated booster [2]. Vaccinating children and adolescents against severe acute respiratory syndrome coronavirus 2 (SARS-CoV-2) can reduce the risk of infection [3–5], complications, and COVID-19 sequelae, including hospitalization [5–7] and multisystem inflammatory syndrome in children (MIS-C) [8, 9]. However, as of January 2024, only 11% of US children have received the latest 2023–2024 COVID-19 booster [10].

mRNA-1273 (SPIKEVAX; Moderna, Inc, Cambridge, MA, USA; containing mRNAs encoding for ancestral SARS-CoV-2 strain S protein), which was developed in response to the SARS-CoV-2 pandemic, is authorized in many countries as a 2-dose primary series for individuals aged 6 months and older [11–13]. In June 2022, the US Food and Drug Administration (FDA) amended its Emergency Use Authorization to include the use of mRNA-1273 in children aged 6 months through 17 years [13]. In children 6 months–11 years, mRNA-1273 authorization was based on findings from the phase 2/3 KidCOVE study (NCT04796896), wherein 2 doses (25 µg for ages 6 months–5 years; 50 µg for ages 6–11 years) were immunogenic and had an acceptable safety profile [3, 4]. Notably, the immunogenicity profile observed after primary series vaccination was consistent with that observed in adolescents and young adults (100-µg doses) in the pivotal efficacy study, and mRNA-1273 was efficacious against COVID-19 in children aged 6 months–11 years [3, 4].

Immunity in adults (≥18 years) wanes over time after SARS-CoV-2 vaccination, including with mRNA-1273 [14–16]. Additionally, following the emergence of SARS-CoV-2 variants, vaccine effectiveness is reduced [16]. An mRNA-1273 booster dose administered to adults 6 months or more after the primary series increased the magnitude and breadth of immune response against SARS-CoV-2 [17, 18] and was effective against COVID-19, including disease caused by the Omicron variant [16]. Based on studies of mRNA-1273 as a primary series in pediatric populations, the benefits observed among boosted adults are similarly expected to accrue to boosted children. Thus, we evaluated the safety, immunogenicity, and efficacy of an mRNA-1273 booster dose administered 6 months or more after primary series completion among children aged 6 months–11 years (NCT04796896).

## METHODS

### Trial Design and Participants

This study enrolled participants aged 6 months–11 years at 80 US and 8 Canadian sites [3, 4]. Eligible children were generally healthy, but those with stable chronic conditions were also included. Inclusion/exclusion criteria are included in the Supplementary Methods. Details on the representativeness of the study participants are available (Supplementary Table 1).

The primary series study design has been described previously [3, 4]. The study was conducted in 2 parts, with an open-label, dose-finding phase (part 1) and an observer-blinded, randomized, placebo-controlled phase (part 2). The present analysis was conducted in participants aged 6 months–5 years (from part 1) and aged 6–11 years (from parts 1 and 2) who received the 2-dose mRNA-1273 primary series at the licensed dose (25 µg [6 months–5 years]; 50 µg [6–11 years]) approximately 28 days apart, and received an optional mRNA-1273 booster dose (10 µg and 25 µg, respectively) 6 months or more after completing the primary series (Supplementary Figure 1). Participants were assigned to age groups based on age at enrollment. Data collection was from the start of the booster phase (March 2022) to the data cutoff date (1 June 2023).

### Vaccine

mRNA-1273 is an mRNA-lipid nanoparticle vaccine encoding for the full-length, 2-proline prefusion stabilized SARS-CoV-2 S protein [19]. mRNA-1273 was provided as a sterile liquid for injection at 0.2 mg/mL, diluted with 0.9% sodium chloride to the appropriate dose, and administered at a 0.5-mL dose volume intramuscularly 6 months or more after dose 2 of the primary series.

### Objectives

The primary safety objective was to evaluate the safety and reactogenicity of an mRNA-1273 booster dose. The primary immunogenicity objective was to infer efficacy of an mRNA-1273 booster by establishing noninferiority of antibody responses after the booster (day 29) in children compared with responses after the mRNA-1273 primary series (day 57) in young adults (18–25 years) enrolled in the pivotal phase 3 study (COVE) [20]. Key secondary objectives are described in the Supplementary Methods.

### Safety Assessments

Safety endpoints were solicited local and systemic adverse reactions (ARs) within 7 days following the booster (see Supplementary Methods); unsolicited adverse events (AEs) within 28 days following the booster; and serious AEs (SAEs), medically attended AEs (MAAEs), AEs leading to discontinuation, or AEs of special interest (AESIs) from the booster to the end of the study.

### Immunogenicity Assessments

Serum samples for immunogenicity analysis were collected at baseline (day 1), day 209 (6 months following completion of the 2-dose primary series), booster day 1 (pre-booster), and 28 days after the booster (day 29); historical serum samples from young adults (COVE) at baseline (day 1) and 28 days after dose 1 (day 29) and dose 2 (day 57) of the primary series were also evaluated. Neutralizing antibody (nAb) geometric mean (GM) concentrations (GMCs) and seroresponse rates (SRRs) against ancestral SARS-CoV-2 with D614G were assessed using a validated pseudo-virus neutralizing assay [21] (Supplementary Methods). SARS-CoV-2 S protein‒specific serum binding antibody (bAb) GM levels against ancestral SARS-CoV-2 and Delta AY.4 or Omicron BA.1 variants were measured by validated Meso Scale Discovery (MSD) multiplex binding antibody assay (Supplementary Methods) [22].

### Statistical Analyses

Sample size determination and study populations are described in the Supplementary Methods. Safety and reactogenicity were descriptively analyzed as counts and percentages among participants in the safety and solicited safety sets, respectively. Primary immunogenicity analyses were only conducted in the per-protocol immunogenicity subset with negative SARS-CoV-2 status at the time of the booster (children) or dose 1 of the primary series (young adult comparison group). Negative SARS-CoV-2 status was defined as having a negative reverse transcriptase–polymerase chain reaction (RT-PCR) test and a negative serology test (based on bAb specific to SARS-CoV-2 nucleocapsid, a nonvaccine antigen, measured by Roche Elecsys Anti-SARS-CoV-2 assay) at the time of the booster (children) or pre-vaccination (young adults). The GMCs of nAb and corresponding 95% confidence intervals (CIs) were estimated using the *t*-distribution. An analysis of covariance (ANCOVA) model was performed to assess the difference between nAb levels after the mRNA-1273 booster in children (day 29) and after the mRNA-1273 primary series in young adults (day 57). The GM ratio (GMR) of nAb levels in children and adults and corresponding 95% CIs were estimated using the geometric least squares means estimated from the ANCOVA model. Noninferiority was declared if the lower bound of the 95% CI of the GMR (GMC post-booster day 29 [children] over GMC post-dose 2 day 57 [young adults]) was greater than 0.667 (or >1/1.5). Noninferiority was declared if the lower bound of the 95% CI of the SRR difference between the 2 groups was 10% or greater. The SRR analyses are described in the Supplementary Methods.

## RESULTS

### 6-Month-Olds to 5-Year-Olds

#### Participants

**Figure 1.**
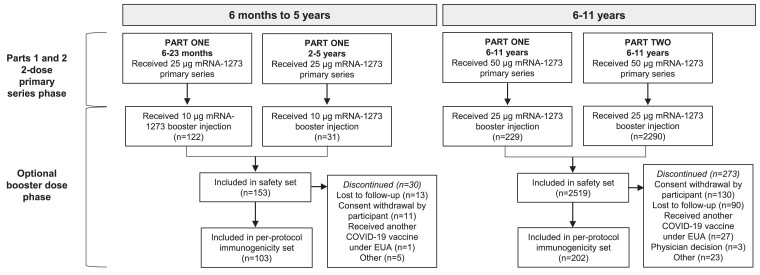
Disposition by age group. Eligible participants who received a prior 2-dose primary series of mRNA-1273 (6 months–5 years: 2 doses of mRNA-1273 25 µg; 6–11 years: 2 doses of mRNA-1273 50 µg) and received a booster dose of mRNA-1273 ≥6 months after the second dose. For the cohort aged 6 months–5 years (n = 153), participants received a 10-µg booster dose of mRNA-1273; those aged 6–11 years (n = 2519) received a 25-µg booster dose of mRNA-1273. Data cutoff date was 1 June 2023. Abbreviations: COVID-19, coronavirus disease 2019; EUA, Emergency Use Authorization.

Overall, 153 participants aged 6 months–5 years received a 25-µg mRNA-1273 primary series and a 10-µg mRNA-1273 booster dose (Figure 1). The median age was 1 year when starting the primary series and 2 years at receipt of booster; 56.2% were male, with 80.4% White, 2.6% Black, 5.9% Asian, and 10.5% Hispanic or Latino participants (Table 1). Approximately 23% of participants had evidence of SARS-CoV-2 infection at the time of boosting. The median time since primary series dose 2 to booster was 306 days.

**Table 1.** Participant Demographics by Age Group (Safety Set)

	mRNA-1273	
	Booster: 10 µg, 6 Months to 5 Years (n = 153)	Booster: 25 µg, 6 to 11 Years (n = 2519)
Age at receipt of booster dose, y		
Mean (SD)	2.7 (1.1)	9.7 (1.7)
Median (IQR)	2 (2–3)	10 (8–11)
Range	1–6	7–13
Age at receipt of booster dose, mo		
Mean (SD)	32.4 (13.3)	…
Median (IQR)	28 (24–35)	…
Range	17–71	…
Sex, n (%)		
Male	86 (56.2)	1330 (52.8)
Female	67 (43.8)	1189 (47.2)
Race, n (%)		
White	123 (80.4)	1657 (65.8)
Black	4 (2.6)	279 (11.1)
Asian	9 (5.9)	203 (8.1)
American Indian or Alaska Native	1 (0.7)	11 (0.4)
Native Hawaiian or Other Pacific Islander	0	4 (0.2)
Multiracial	12 (7.8)	291 (11.6)
Other	4 (2.6)	49 (1.9)
Not reported	0	21 (0.8)
Unknown	0	4 (0.2)
Ethnicity, n (%)		
Hispanic or Latino	16 (10.5)	425 (16.9)
Not Hispanic or Latino	136 (88.9)	2072 (82.3)
Not reported	1 (0.7)	15 (0.6)
Unknown	0	7 (0.3)
Pre-booster SARS-CoV-2 status,^a^ n (%)		
Negative	98 (64.1)	1267 (50.3)
Positive	35 (22.9)	1060 (42.1)
Missing	20 (13.1)	192 (7.6)

Abbreviations: IQR, interquartile range; RT-PCR, reverse transcriptase–polymerase chain reaction; SARS-CoV-2, severe acute respiratory syndrome coronavirus 2.

^a^Pre-booster SARS-CoV-2 status: negative status was defined as having a negative RT-PCR test and negative serology test (based on binding antibody specific to SARS-CoV-2 nucleocapsid as measured by Roche Elecsys Anti-SARS-CoV-2 assay) at the date of the booster dose. Positive status was defined as having either a positive RT-PCR test or positive serology (based on binding antibody specific to SARS-CoV-2 nucleocapsid as measured by Roche Elecsys Anti-SARS-CoV-2 assay) on the date of the booster dose.

#### Safety

**Figure 2.**
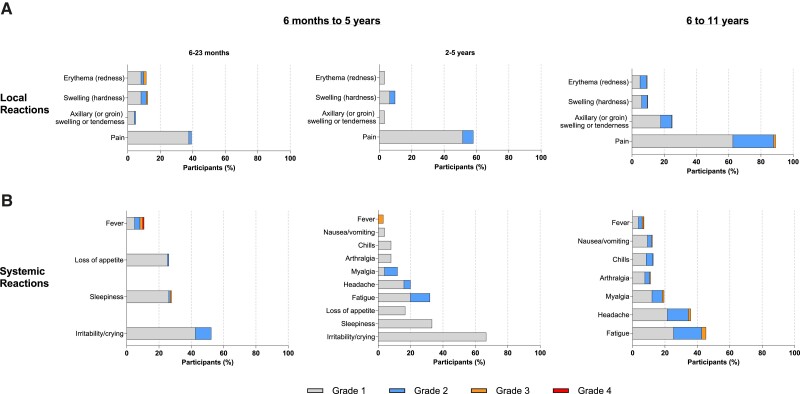
Local reactions and systemic events after booster vaccination by age group. The percentage of participants reporting local (*A*) or systemic (*B*) events by grade within 7 days of receiving a booster dose of mRNA-1273. Numbers of participants derived from the solicited safety set: 6 –23 months, n = 122; 2–5 years, n = 31; 6–11 years, n = 2487.

Solicited local ARs within 7 days after booster vaccination occurred in 75 of 153 participants (49.0%). Pain was the most common local AR (43.1%) (Figure 2). Most solicited local ARs were mild to moderate, with 2 grade 3 events (1.3%; 2 events of erythema [redness] and 1 event of swelling [hardness] in 2 participants; both aged 6–23 months) and no grade 4 events reported. Solicited systemic ARs within 7 days after booster occurred in 97 of 153 participants (63.4%) and were mostly mild to moderate; 5 participants (3.3%) had grade 3 or higher systemic ARs (1 [0.8%] grade 3 sleepiness, 3 [2.0%] grade 3 fever [39.0°–40.0°C], and 1 [0.7%] grade 4 fever [>40°C]). Irritability/crying (53.1%) was the most common systemic AR. The grade 4 event occurred in a participant who was SARS-CoV-2–negative at baseline in the 6–23-month age group. The participant experienced initial fever to 38.5°C (101.3°F) 3 days after the booster but was otherwise well; their temperature reached a maximum of 40.5°C (105.0°F) on day 5 after the booster.

Unsolicited AEs within 28 days after the booster occurred in 39 of 153 participants (25.5%); 5 (3.3%) were considered by the investigator to be related to vaccination (reactogenicity events of vomiting [n = 1] and general disorders and administration site conditions [n = 4]) (Table 2). At data cutoff (1 June 2023), the median (interquartile range [IQR]) duration of safety follow-up after the booster was 364 (331–374) days. An MAAE was experienced by 87 participants (56.9%); all were assessed as being unrelated to vaccination. One participant (0.7%) experienced an SAE (streptococcal infection with pharyngeal abscess), which was assessed as being unrelated to vaccination. No severe AEs, deaths, or AEs leading to study discontinuation were reported. Two participants (1.3%) experienced an AESI (epilepsy on day 138 and erythema multiforme on day 157); both were assessed as being unrelated to vaccination. No cases of myocarditis or pericarditis were reported.

**Table 2.** Summary of Unsolicited Adverse Events After Booster Vaccination by Age Group (Safety Set)

	mRNA-1273	
	Booster: 10 µg, 6 Months to 5 Years (n = 153)	Booster: 25 µg, 6 to 11 Years (n = 2519)
All unsolicited AEs within 28 d, n (%)	39 (25.5)	374 (14.8)
All unsolicited AEs to data cutoff date (1 June 2023), n (%)	97 (63.4)	1152 (45.7)
SAEs	1 (0.7)	11 (0.4)
Fatal AEs	0	0
MAAEs	87 (56.9)	1053 (41.8)
AEs leading to study discontinuation	0	0
Severe	0	17 (0.7)
AESIs	2 (1.3)	12 (0.5)
Related^a^ unsolicited AEs within 28 d, n (%)	5 (3.3)	83 (3.3)
All related^a^ unsolicited AEs to data cutoff date (1 June 2023), n (%)	5 (3.3)	84 (3.3)
SAEs	0	0
Fatal AEs	0	0
MAAEs	0	20 (0.8)
AEs leading to study discontinuation	0	0
Severe	0	8 (0.3)
AESIs	0	0

The safety set included all participants who received an mRNA-1273 booster dose.

Abbreviations: AE, adverse event; AESI, adverse event of special interest; MAAE, medically attended adverse event; SAE, serious adverse event.

^a^The investigator considered that there was a reasonable possibility of a relationship with the study vaccine.

#### Immunogenicity

**Figure 3.**
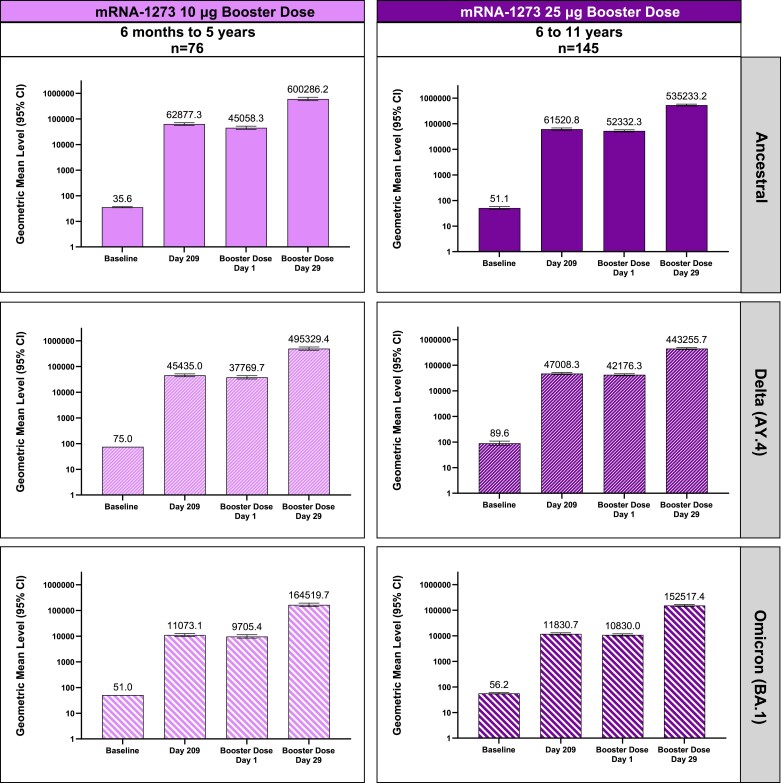
Binding antibody levels against ancestral SARS-CoV-2, Delta (AY.4) variant, and Omicron BA.1 variant after booster vaccination among children aged 6 months–11 years with pre-booster SARS-CoV-2–negative status (per-protocol immunogenicity, SARS-CoV-2–negative set). Binding antibody responses were assessed at baseline (day 1), day 209 following primary series vaccination, booster dose day 1 (pre-booster), and booster day 29 among participants with no evidence of current or prior SARS-CoV-2 infection at the pre-booster visit (n = 76, 6 months–5 years; n = 145, 6–11 years). Pre-booster SARS-CoV-2–negative status was defined as having a negative RT-PCR test and negative serology test (based on binding antibody specific to SARS-CoV-2 nucleocapsid as measured by Roche Elecsys Anti-SARS-CoV-2 assay) at the date of the booster dose. Abbreviations: CI, confidence interval; RT-PCR, reverse transcriptase–polymerase chain reaction; SARS-CoV-2, severe acute respiratory syndrome coronavirus 2.

Regardless of pre-booster SARS-CoV-2 status, mRNA-1273 booster administration induced measurable increases in nAb levels relative to pre-booster levels among participants aged 6 months–5 years (Supplementary Figure 2). Among participants with SARS-CoV-2–negative status at the pre-booster visit (n = 76), a booster dose increased observed nAb GMCs (95% CI) against ancestral SARS-CoV-2 by 16-fold, increasing from 341 (284–409) at pre-booster day 1 to 5457 (4526–6580) at day 29 post-booster (Table 3). The ANCOVA-modeled GMR of nAb GMCs after the booster compared with levels observed after the primary series in young adults was 3.9 (95% CI: 3.2–4.8), meeting noninferiority criteria. The SRR was 100% (72/72) at day 29 among participants with pre-booster SARS-CoV-2–negative status. The SRR difference at day 29 also met the noninferiority criteria (0.7%; 95% CI: −4.4% to 2.4%). Among participants with SARS-CoV-2–positive status at the pre-booster visit (n = 20), there was a 3-fold increase in observed GMCs (95% CI) from 3474 (1858–6497) at pre-booster day 1 to 11 328 (8578–14 958) at day 29 post-booster (Supplementary Figure 2). Additionally, relative to pre-booster day 1 levels, bAb GM levels increased after the booster against ancestral SARS-CoV-2, Delta AY.4, and Omicron BA.1 among participants with pre-booster SARS-CoV-2–negative status (Figure 3).

**Table 3.** Serum Neutralizing Antibody Levels Against Ancestral SARS-CoV-2 After Booster Vaccination (Per-Protocol Immunogenicity, SARS-CoV-2–Negative Set)

	Children Aged 6 Months to 5 Years, mRNA-1273 Booster (10 µg) (n = 76)	Children Aged 6 to 11 Years, mRNA-1273 Booster (25 µg) (n = 145)	Young Adults Aged 18 to 25 Years, mRNA-1273 Primary Series (100 µg) (n = 296)
Day 57 post-primary series, n	55	58	294
Observed GMC (95% CI)^a^	1431.2 (1201.2–1705.2)	1533.1 (1318.3–1782.9)	1400.4 (1272.7–1541.0)
Baseline (pre-booster day 1 or pre-dose 1^b^), n	72	144	295
Observed GMC (95% CI)^a^	340.6 (283.7–408.8)	434.1 (389.5–483.9)	11.1 (10.5–11.6)
Day 29 post-booster, n	76	145	…
Observed GMC (95% CI)^a^	5457.2 (4525.7–6580.3)	5561.0 (5036.3–6140.3)	…
GMFR (95% CI)^c^	15.8 (12.8–19.4)	12.8 (11.3–14.6)	…
GMR day 29 post-booster children versus day 57 young adults (95% CI)^d^	3.897 (3.158–4.808)	3.971 (3.409–4.626)	…
Day 29 post-booster or day 57 post-primary SRR,^e^ n/N (%) [95% CI]	72/72 (100) [95.0–100]	137/137 (100) [97.3–100]	292/294 (99.3) [97.6–99.9]
SRR difference vs young adults,^f^ % (95% CI)	0.7 (−4.4 to 2.4)	0.7 (−2.1 to 2.4)	…

Numbers of participants in the per-protocol immunogenicity subset with pre-booster SARS-CoV-2–negative status in KidCOVE, or the per-protocol immunogenicity subset for the primary series in the COVE trial, are shown.

Abbreviations: ANCOVA, analysis of covariance; CI, confidence interval; GM, geometric mean; GMC, geometric mean concentration; GMFR, geometric mean fold rise; GMR, geometric mean ratio; LLOQ, lower limit of quantification; LS, least squares; SARS-CoV-2, severe acute respiratory syndrome coronavirus 2; SRR, seroresponse rate.

^a^95% CIs were calculated using the *t*-distribution of the log-transformed values or the difference in the log-transformed values for GMC and then back-transformed to the original scale for presentation.

^b^Pre-booster day 1 in children aged 6 months to 5 years and 6 to 11 years; pre-dose 1 in young adults aged 18 to 25 years.

^c^GMFR refers to the fold rise in GMC at the 2 defined time points and was calculated as booster day 29/pre-booster day 1. The 95% CIs were calculated using the *t*-distribution of the log-transformed values or the difference in the log-transformed values, then back-transformed to the original scale for presentation of GMC or GMFR 95% CIs.

^d^Log-transformed antibody levels were analyzed using an ANCOVA model with the age group variable (children and young adults) as a fixed effect. The resulting LS means, difference of LS means, and 95% CIs were back-transformed to the original scale for presentation of GMC and GMR with 95% CIs.

^e^The SRR at day 29 from baseline (pre-dose 1 of the primary series) was defined as the percentage of participants with a change from below LLOQ to equal or above 4× LLOQ, or at least a 4-fold rise if the baseline was ≥LLOQ. Corresponding 95% CIs were calculated using the Clopper-Pearson method.

^f^The SRR difference 95% CI was calculated using the Miettinen-Nurminen (score) confidence limits.

### 6- to 11-Year-Olds

#### Participants

Overall, 2519 participants aged 6–11 years received a 25-µg mRNA-1273 booster dose (Figure 1). The median age was 8 years when starting the primary series and 10 years at receipt of booster; 52.8% were male, with 65.8% White, 11.1% Black, 8.1% Asian, and 16.9% Hispanic or Latino participants (Table 1). Approximately 42% of participants had evidence of SARS-CoV-2 infection at the time of boosting. The median time since the primary series dose 2 to booster was 235 days.

#### Safety

Solicited local ARs within 7 days after booster vaccination were reported by 2243 of 2487 participants (90.3%). Most local ARs were mild to moderate, with grade 3 events reported by 48 participants (1.9%); no grade 4 events were reported. The most common local ARs were pain (89.3%) and axillary node swelling/tenderness (25.0%) (Figure 2). Solicited systemic ARs within 7 days after booster were reported by 1523 of 2487 participants (61.3%) and were mostly mild to moderate, with 114 (4.6%) reporting grade 3 events and 2 (<0.1%) reporting grade 4 events. Both grade 4 events were fever, reported in 2 participants with concurrent symptomatic COVID-19. The most common (>20%) systemic ARs were fatigue (45.4%) and headache (36.0%).

Unsolicited AEs within 28 days after the booster were reported by 374 of 2519 of participants (14.8%); 83 (3.3%) were considered by the investigator to be related to mRNA-1273 (Table 2). Adverse events were most often classified as infections/infestations (8.2% [207/2519 participants]). At data cutoff (1 June 2023), the median (IQR) duration of safety follow-up after the booster dose was 363 (337–371) days and 1053 participants (41.8%) had experienced MAAEs; 20 (0.8%) experienced an MAAE assessed by the investigator as being vaccine-related (Supplementary Table 2). Seventeen participants (0.7%) reported severe AEs and 8 (0.3%) were considered by the investigator to be vaccine-related (Supplementary Table 2). Eleven participants (0.4%) experienced SAEs; none were considered vaccine-related (Table 2). There were no deaths or AEs leading to study discontinuation. Twelve participants (0.5%) had AESIs; none were considered vaccine-related (Supplementary Table 3). No cases of myocarditis or pericarditis were reported.

#### Immunogenicity

Regardless of pre-booster SARS-CoV-2 status, an mRNA-1273 booster induced increases in nAbs relative to pre-booster levels among participants aged 6–11 years (Supplementary Figure 3). Among participants with SARS-CoV-2–negative status at the pre-booster visit (n = 145), an mRNA-1273 booster increased observed nAb GMCs (95% CI) against ancestral SARS-CoV-2 by 13-fold, from 434 (390–484) at pre-booster day 1 to 5561 (5036–6140) at day 29 post-booster (Table 3). The ANCOVA-modeled GMR of nAb GMCs after the booster compared with levels after the primary series in young adults was 4.0 (95% CI: 3.4–4.6), meeting the noninferiority criterion. The SRR was 100% (137/137) at day 29 among participants with SARS-CoV-2–negative status. The SRR difference of 0.7% (95% CI: −2.1% to 2.4%) also met the noninferiority criterion. Among participants with SARS-CoV-2–positive status at the pre-booster visit (n = 48), there was a 2-fold increase in nAb GMCs (95% CIs) from 4318 (3333–5595) at pre-booster day 1 to 8387 (6980–10 077) at day 29 post-booster (Supplementary Figure 3). Additionally, relative to pre-booster day 1 levels, a booster dose increased bAb GM levels against ancestral SARS-CoV-2, Delta AY.4, and Omicron BA.1 among participants with SARS-CoV-2–negative status (Figure 3).

## DISCUSSION

An mRNA-1273 booster dose administered to children aged 6 months–5 years (10 µg) or 6–11 years (25 µg) had a consistent safety profile across age groups, and elicited immune responses against the SARS-CoV-2 ancestral strain that were noninferior to responses in young adults following primary series vaccination [20]. As of February 2024, approximately 79 000 COVID-19–confirmed hospitalizations have been reported in US children aged 11 years and younger, with approximately 120 weekly admissions during the 2023–2024 seasonal peak, highlighting the continued need to protect against severe illness through booster vaccination [23].

The mRNA-1273 booster had a reactogenicity profile similar to that of the primary series [3, 4]. Unsolicited AEs after the booster were similar in profile to unsolicited AEs reported after the primary series (most commonly infections/infestations), and there were no vaccine-related SAEs reported in either age stratum. Although a greater number of participants aged 6–11 years experienced vaccine-related AEs and severe AEs compared with those aged 6 months–5 years, this is likely due to differing vaccine group sizes, differing follow-up periods, and age-related variation in the reporting of reactogenicity. All vaccine-related severe AEs were reactogenicity events. There were no identified cases of MIS-C or cases suggestive of myocarditis/pericarditis, and no participants withdrew from the study due to AEs after booster dose receipt. Overall, findings were consistent with the known safety profile of mRNA-1273.

An mRNA-1273 booster dose increased serum nAbs in children regardless of SARS-CoV-2 serostatus. These findings suggest that participants with seropositive status also derive an immunological benefit from booster vaccination. Notably, in participants with SARS-CoV-2–negative status, nAb responses were noninferior to those observed in young adults in the pivotal COVE study, where vaccine efficacy against COVID-19 was established [20], and it is anticipated that a booster dose in children will confer similar benefits to those seen in adults who received a booster dose in previous clinical [17] and observational [16] studies. Additionally, an mRNA-1273 booster dose increased serum bAb levels against ancestral SARS-CoV-2 and, to a lesser extent, against Omicron BA.1 and Delta AY.4 in children. Studies in adults have demonstrated that an mRNA-1273 booster dose can restore the immune decline observed over time after primary vaccination and enhance effectiveness against Delta and Omicron variants [16, 17, 24]. Notably, in this study, bAb responses remained high 6 months after the primary series, which may have implications for booster dose timing. Furthermore, serum nAbs increased regardless of the median time interval between the second injection of the mRNA-1273 primary series and the booster dose, which ranged from 8 to 10 months. Similar results were observed in the COVE study in adults, which showed consistent post-booster nAb levels regardless of the time interval (12–16 months) between the mRNA-1273 primary series and the booster dose [25].

The limited sample size of the study precluded the detection of rare safety events, such as MIS-C or myocarditis/pericarditis. The scope of overall exposure data across the mRNA-1273 pediatric development program helps support and supplement the relatively small number of participants in this booster study (∼3000 infants and children received a booster dose of mRNA-1273). Reassuringly, analyses of post-marketing data (Vaccine Adverse Event Reporting System and Vaccine Safety Datalink) in children aged 6 months–11 years found no statistically significant signals for myocarditis/pericarditis [26, 27]. The benefits of preventing COVID-19 and the subsequent sequelae in children outweigh the risks from exposure to an mRNA-1273 booster dose, which is supported by the safety findings here and across the clinical studies and post-authorization experience.

In conclusion, an mRNA-1273 booster has a consistent safety profile across age groups and elicited immune responses, supporting administration of a booster dose in children aged 6 months–11 years. These data, in conjunction with the clinical data of mRNA-1273 boosters in adolescents and adults, as well as data on variant-containing mRNA-1273 boosters in adults, contributed to recent US Advisory Committee on Immunization Practices recommendations for the administration of a monovalent XBB variant–containing COVID-19 mRNA vaccine to all eligible persons aged 6 months and older [28].

## Supplementary Data

Supplementary materials are available at *Clinical Infectious Diseases* online. Consisting of data provided by the authors to benefit the reader, the posted materials are not copyedited and are the sole responsibility of the authors, so questions or comments should be addressed to the corresponding author.

## Supplementary Material

ciae420_Supplementary_Data

## Notes

***Author contributions.*** V. B., C. B. C., L. d. L., W. D., H. Z., K. S., E. J. A., R. D., J. M., and S. S. G. all contributed to the concept and design of the study. Data collection was performed by V. B., C. B. C., C. A. Rostad, Q. C., L. d. L., M. D., A. G., D. J., S. N., S. P., M. R., C. A. Rodriguez, K. L., W. T., M. B., B. G., and E. J. A. Analysis and interpretation of data were carried out by V. B., C. B. C., C. A. Rostad, A. Y., M. B., X. Z., W. D., W. X., H. Z., B. G., R. K., K. S., E. J. A., R. D., J. M., and S. S. G. All authors provided intellectual contribution for the writing and review of the manuscript. All authors approved the final draft of the manuscript.

***Acknowledgments.*** The authors thank the children and their families for their participation in this clinical trial, and they are grateful to the research staff who contributed to conduct of the trial. Medical writing and editorial assistance (including the first draft under the guidance of authors) were provided by Andy Kerr, PhD, Ashlea Inan, PhD, Kate J. Russin, PhD, and Renee Gordon, PhD, of MEDiSTRAVA in accordance with Good Publication Practice guidelines, funded by Moderna, Inc, and under the direction of the authors.

***Financial support*.** This work was supported, in whole or in part with federal funds, by the United States Department of Health and Human Services, Administration for Strategic Preparedness and Response, Biomedical Advanced Research and Development Authority, under contract no. 75A50120C00034; the National Institute of Allergy and Infectious Diseases, part of the National Institutes of Health (grant numbers UM1AI148576, UM1AI148452, UM1AI148689, UM1AI148450, UM1AI148372, and UM1AI148575); and the National Institutes of Health, National Center for Advancing Translational Sciences (grant number UL1TR001453).

***Data availability.*** Access to patient-level data presented in the article (immunogenicity, safety, and reactogenicity) and supporting clinical documents by qualified external researchers who provide methodologically sound scientific proposals may be available upon reasonable request and subject to review once the trial is complete. Such requests can be made to Moderna, Inc, 325 Binney St, Cambridge, MA 02142. A materials transfer and/or data access agreement with the sponsor will be required for accessing shared data. All other relevant data are presented in the paper.

***Ethics statement.*** The protocol and other relevant documents were approved by the central institutional review board (Advarra, Inc) and the study was conducted in accordance with the protocol, applicable laws and regulatory requirements, the International Council for Harmonization, Good Clinical Practice guidelines, and the ethical principles derived from the Declaration of Helsinki and Council for International Organizations of Medical Sciences International Ethical Guidelines. Participants' parent(s)/legally authorized representative(s) provided written informed consent and the participant, as applicable, signed the assent form before conduct of study procedures.
