# Supplementary material for: Safety and Immunogenicity of an mRNA-1273 Booster in Children
Source: Clin Infect Dis. 2024 Aug 19;79(6):1524–32. doi: 10.1093/cid/ciae420 (PMC11650855; doi:10.1093/cid/ciae420)
Supplement: ciae420_Supplementary_Data [file ciae420_supplementary_data.docx]

# SUPPLEMENTARY APPENDIX

Supplement to: Berthaud V, Creech B, Rostad CA, et al. Safety and Immunogenicity of an mRNA-1273 Booster in Children.

**Table of Contents**

[List of KidCOVE Investigators 2](#_Toc173509837)

[Supplementary Methods 7](#_Toc173509838)

[Inclusion and Exclusion Criteria 7](#_Toc173509839)

[Key Secondary Objectives 9](#_Toc173509840)

[Solicited Adverse Reactions 9](#_Toc173509841)

[Pseudovirus Neutralizing Assay 9](#_Toc173509842)

[MSD Binding Antibody Assay 10](#_Toc173509843)

[Sample Size 10](#_Toc173509844)

[Analysis Populations 11](#_Toc173509845)

[Seroresponse Rate Analyses 11](#_Toc173509846)

[Supplementary Tables 12](#_Toc173509847)

[Table S1. Background information on COVID-19 and the representativeness of study participants 12](#_Toc173509848)

[Table S2. Participants with vaccine-related MAAEs and related severe AEs by age group throughout the entire study period (to June 1, 2023, data cutoff) 14](#_Toc173509849)

[Table S3. Participants with AESIs by age group throughout the entire study period (to June 1, 2023, data cutoff) 16](#_Toc173509850)

[Supplementary Figures 17](#_Toc173509851)

[Figure S1. Overview of the study design 17](#_Toc173509852)

[Figure S2. Serum neutralizing antibody levels against ancestral SARS-CoV-2 after booster vaccination by SARS-CoV-2 infection status among children 6 months to 5 years (per-protocol immunogenicity set) 18](#_Toc173509853)

[Figure S3. Serum neutralizing antibody levels against ancestral SARS-CoV-2 after booster vaccination by SARS-CoV-2 infection status among children 6-11 years (per-protocol immunogenicity set) 19](#_Toc173509854)

[Supplementary References 20](#_Toc173509855)

List of KidCOVE Investigators

| **Affiliation** | **Principal Investigator** | **Study Group** | **Location** |
| --- | --- | --- | --- |
| Dr. Anil K. Gupta Medicine Professional Corporation | Anil K. Gupta, MD | Bhavya Gandhi  Shrey Acharya | Ontario, Canada |
| University of Alabama at Birmingham/ Children’s of Alabama | Swetha Pinninti, MD | Suresh Boppana, MD  Misty Purser Latting Sama Halima | Birmingham, AL |
| Clinical Research Institute, Inc. | Gary Berman, MD | Krista Sullivan  Amy Roehl  Heidi Olson | Minneapolis, MN |
| Velocity Clinical Research – Salt Lake City – Advanced Clinical Research Jordan Valley | Barbara Rizzardi, MD | Barbara Rizzardi, MD  Vanesa Abad  Lori Luth | West Jordan, UT |
| UMass Memorial Medical Center | Katherine Luzuriaga, MD | John L. Sullivan, MD  Jesica Pagano-Therrien, PhD | Worcester, MA |
| Velocity Clinical Research - Boise | Mark Turner, MD | Mark Turner, MD  Nick Tuttle  Audra Weslowski | Meridian, ID |
| Child Healthcare Associates - East Syracuse | Leonard Weiner, MD | Leonard B. Weiner, MD  Kristen Fluno  Lori J. Ferguson | East Syracuse, NY |
| Vanderbilt University Medical Center | C. Buddy Creech, MD, MPH | Stephanie Rolsma  Shannon Walker  Katherine Sokolow | Nashville, TN |
| Stony Brook University Medical Center  Stony Brook Children’s | Sharon Nachman, MD | Barsha Chakraborty  Jennifer Russell | Stony Brook, NY |
| PI Coor Clinical Research, LLC | Fredric B. Garner, MD | Fredric B. Garner, MD  Joseph P. Balsamo  Sarah O. Crespo | Burke, VA |
| Certified Research Associates | Christopher A. Smith, MD | Christopher A. Smith, MD Danielle Allen  Melinda Morgan | Cortland, NY |
| Ann and Robert H. Lurie Childrens Hospital of Chicago | William J. Muller, MD, PhD | William J. Muller, MD, PhD  Laura Fearn  Sarah Berlatsky | Chicago, IL |
| Emory University School of Medicine | Evan J. Anderson, MD | Christina A. Rostad, MD  Satoshi Kamidani, MD  Lisa Macoy, PNP | Atlanta, GA |
| Children's Hospital of Pittsburgh UPMC University Center | Judith M. Martin, MD | Judith M. Martin, MD  Alejandro Hoberman  Gysella B. Muniz | Pittsburgh, PA |
| Victoria Clinical Research Group | John Clinton, MD | John Clinton, MD  Evelyn Thomas  Kimberly Bundy | Port Lavaca, TX |
| Javara Inc./Privia Medical Group, LLC (Chevy Chase) - Javara  Capitol Medical Group | Daniel Finkelstein, MD | Ana Merkovic  Rupert Vallarta  Shuchismita Bhatt | Chevy Chase, MD |
| Prohealth Research Center | David F. Jativa, MD | Marcela Jativa  Johanna Garcia | Doral, FL |
| Palmetto Pediatrics | Michael L. Leonardi, MD | Michael L. Leonardi, MD  J Bruce Etheridge, MD  Sandi B. [Judkins, RMA](mailto:Judkinssandi.judkins@palmettopediatrics.com) | North Charleston, SC |
| Children’s Hospital Colorado | Myron Levin, MD | Tori Rutherford  Julianne Randlemon  Deina Barton | Aurora, CO |
| Javara Inc. – Dallas | Clifford W. Yut, MD | Clifford W. Yut, MD  Thipsavanh Douangboupha  Jenny Pleitez | The Woodlands, TX |
| The University of New Mexico School of Medicine  The University of New Mexico Hospital | Walter Dehority, MD, MSc | Walter Dehority, MD, MSc Hengameh H. Raissy  Todd Chandler | Albuquerque, NM |
| Children’s Hospital of Philadelphia | Jeffrey S. Gerber, MD, PhD | Jeffrey S. Gerber, MD, PhD Rasheeda Lawler  William R. Otto | Philadelphia, PA |
| Washington University in St. Louis | Jason G. Newland, MD | Jason G. Newland, MD  David A Hunstad  Carol Kao | St. Louis, MO |
| Pennington Biomedical Research Center | Daniel S. Hsia, MD | Daniel S. Hsia, MD  Amy N. Thomassie  Claire Hazlett | Baton Rouge, LA |
| Velocity Clinical Research – Banning | Judith L. Kirstein, MD | Judith L. Kirstein  Hanna He  Julie Kasarjian | Banning, CA |
| Baylor College of Medicine | Erin G. Nicholson, MD | Erin G. Nicholson, MD  C. Mary Healy  Hana M. El Sahly | Houston, TX |
| University of Rochester Medical Center | Mary T. Caserta, MD | Jennifer Nayak  Michell Heiman  Noya Rackovsky | Rochester, NY |
| Cincinnati Children’s Hospital Medical Center | Grant C. Paulsen, MD | Grant C. Paulsen, MD  Robert Frenk  Jennifer Whitaker | Cincinnati, OH |
| Kaiser Permanente Los Angeles Medical Center | William J. Towner, MD | Randi Kendrick  Marissa Barron Gloricel Hernandez-Reyes | Los Angeles, CA |
| University of Wisconsin – Madison University of Hospital and UW Health Clinics | William Hartman, MD, PhD | William Hartman, MD  James Conway  Sheryl Henderson | Madison, WI |
| Henry Ford Health System  Henry Ford Hospital | Marcos Zervos, MD | Marcos Zervos, MD  Mayur Ramesh  Charles Barone | Detroit, MI |
| UCSD Altman Clinical and Translational Research Institute | Stephen A. Spector, MD | Stephen A. Spector, MD  Amaran Moodley  Megan Loughran | La Jolla, CA |
| Texas Center for Drug Development, Inc DM Clinical Research | Kashif R. Ali, MD | Kashif R. Ali, MD  Bonnie Elizabeth Colville  Berenice Ferrero | Houston, TX |
| Cyfair Clinical Research Center | Khozema A. Palanpurwala, MD | Khozema A. Palanpurwala, MD Amy Starr  Christina Schmitt | Houston, TX |
| ACRC Trials | Madhavi Ampajwala, MD | Madhavi Ampajwala, MD  Heema Marwah  Jacob Meadors | Frisco, TX |
| Carey Chronis MD Pediatric, Infant  and Adolescent Medicine | Carey Chronis, MD | Carey Chronis, MD  Rocio Ma  Anthony Sana | Ventura, CA |
| West Houston Clinical Research Service | Oscar De Valle, MD | Oscar De Valle, MD  Nixcela Ares Valdes | Houston, TX |
| Emmaus Research Center, Inc. | Elizabeth Reyes, MD | Elizabeth Reyes, MD  Filipinas Vitug | Anaheim, CA |
| Javara, Inc. – Dallas | Ronald Blair, MD | Robin Scurlark David Martinez  Samantha Villa | Dallas, TX |
| Tanner Clinic | Nathan G. Forbush, MD | Nathan G. Forbush, MD  Candace K. Smith  Adam M. Hutchinson, Sr. | Layton, UT |
| Velocity Clinical Research – Providence | Richard K. Ohnmacht, MD | Richard K. Ohnmacht, MD  Lynne Haughey  Ariana Stanton | Warwick, RI |
| Alliance for Multispecialty Research – El Dorado | Michael A. Rausch, MD | Michael A. Rausch, MD  Ariel Welty  Melissa Burton | El Dorado, KS |
| Pininos Pediatric Services | Jorge G. Sainz, MD | Jorge G. Sainz, MD  David Leiman  Harold Minkowitz | El Paso, TX |
| Coastal Pediatric Associates | Robert A. Clifford, MD | Jordan Hitchcock  Christine Butler  Lisanne Zharsky | Charleston, SC |
| Allied Biomedical Research Institute | Michael M. Pfeffer, MD | Michael M. Pfeffer, MD | Miami, FL |
| University of Maryland School of Medicine | James D. Campbell, MD, MS | E. Adrianne Hammershaimb, MD Andrea A. Berry, MD  Ginny Cummings, CRNP | Baltimore, MD |
| The Pediatric Center of Frederick | James D. Campbell, MD, MS | E. Adrianne Hammershaimb, MD Andrea A. Berry, MD  Ginny Cummings, CRNP | Frederick, MD |
| Medical Center of South Carolina | Andrew Atz, MD | Natasha M. Ruth  John M. Costello  Kreighton Milks | North Charleston, SC |
| Family Medical Clinic | Sreedhar Chava, MD | Sreedhar Chava, MD  Nichole Lopez  Monica Lopez | El Monte, CA |
| University of Florida Jacksonville | Mobeen Rathore, MD | Erin Zornes  Jamilah Tejan | Jacksonville, FL |
| Quality Clinical Research | Michael Dunn, MD | Saneetria Shannon  Elizabeth Olan  Melissa Frampton | Omaha, NE |
| IResearch Atlanta, LLC | Kimball A. Johnson, MD | Kimball A. Johnson, MD  Heather Beitz  Joy Becker | Decatur, GA |
| Meridian Clinical Research, LLC (Hastings- Nebraska) | Daniel J. Leonard, DO | Daniel J. Leonard, DO  Kristine Johnson  Dionna Gydesen | Hastings, NE |
| OnSite Clinical Solutions, LLC | Arin Piramzadian, DO | Arin Piramzadian, DO  Marriah Decosta  Brianna Bruggeman | Charlotte, NC |
| Kissimmee Clinical Research (KCR) | Konda M. Reddy, MD | Konda M. Reddy, MD  Maria Reynoso  Nilsa Cruz | Kissimmee, FL |
| Clinical Research Partners, LLC | Richard L. Bennett, Jr., MD | Richard L. Bennett, Jr., MD Robert Call  Banks Tuner | Richmond, VA |
| MedPharmics, LLC. – Albuquerque | Quito Carr, MD | Nicol Chavez  Maria Rascon | Albuquerque, NM |
| University of Missouri Health Care System University of Missouri School of Medicine | Christelle Ilboudo, MD | Christelle Ilboudo, MD  Amruta Padhye  Britlyn Brown | Columbia, MO |
| Tufts Medical Center | Andrew Siesennop, MD | Andrew Siesennop, MD  Brian Chow  Vidya Iyer | Boston, MA |
| BRCR Global Texas | Ivan Melendez, MD | Athanasi Orfanos  Braulio Vizcayno  Carlos Del Ángel | Edinburg, TX |
| Meharry Medical College – Division of Infectious Diseases, Clinical and Translational Research Center | Vladimir Berthaud, MD, MPH | Xylina D. Bean  Shahana Choudhury  Christopher J. Keefer | Nashville, TN |
| Our Lady of the Lake Regional Medical Center  Our Lady of the Lake Children’s Health | Michael Bolton, MD | Michael Bolton, MD  JoAnne Hollingsworth  Micah Klumpp | Baton Rouge, LA |
| Pediatric Associates of Fall River | Walter J. Rok, MD | Walter J. Rok, MD  Theresa Ferland  Anne Marie Rezendes | Fall River, MA |
| Pensacola Research Consultants Inc. Avanza Medical Research Center | Randall Reese, MD | Jami Steinmetz  Carol Andrews  David Hensley | Pensacola, FL |
| University of South Florida,  Morsani College of Medicine  John S. Curran, MD, Children’s Health Center | Carina A. Rodriguez, MD | Reed Ryan  Amanda Morton  Alicia Marion | Tampa, FL |
| Tulane Medical Center  Tulane University School of Medicine | Monika Dietrich, MD | Leslie Smitley  Deidra Pierre  Monique Diles | New Orleans, LA |
| University of Kentucky Healthcare – Turfland | George J. Fuchs, MD | George J. Fuchs, MD  John Bauer | Lexington, KY |
| Crossroads Clinical Research (Victoria) | Richard H. Leggett, DO | Richard H. Leggett, DO Debbie Gerloff  Eric Castillo | Victoria, TX |
| University of Minnesota Masonic Children’s Hospital  University of Minnesota Medical Center | Shane McAllister, MD, PhD | Shane McAllister, MD, PhD  Jill Foster  Gwen Fischer | Minneapolis, MN |
| MediSync Clinical Research Hattiesburg Clinic | Rambod Rouhbakhsh, MD, MBA | Rambod Rouhbakhsh, MD, MBA John Johnson  Kenneth Gooch | Petal, MS |
| Yale University School of Medicine – Church Street Research Unit | Inci Yildirim, MD, PhD | Inci Yildirim, MD, PhD  Heidi Zapata  Kelly Anastasio | New Haven, CT |
| Lynn Health Science Institute | Carl P. Griffin, MD | Carl P. Griffin, MD  Chalimar Rojo  Dalia Tovar | Oklahoma City, OK |
| Massachusetts General Hospital | Wayne Shreffler, MD, PhD | Wayne Shreffler, MD, PhD  Lael Yonker  MGH Shrefflab Clinical Research Team | Boston, MA |
| Meridian Clinical Research  (Norfolk-Nebraska) | Keith Vrbicky, MD | Keith Vrbicky, MD  Charles Harper  Chelsie Nutsch | Norfolk, NE |
| Meridian Clinical Research  (Endwell-New York) | Frank S. Eder, MD | Frank S. Eder, MD  Abigail Wine  Carolyn Grausgruber | Binghamton, NY |
| Center for Clinical Trials, LLC | Liberation B. De Leon, MD | Joseph Anthony  Villanueva Vasco  Catalina Villanueva | Paramount, CA |
| Tekton Research – Texas | Paul K. Pickrell, MD | Hussain Shabbir Malbari, MD  Carol Ann Linebarger  Rita Pokuaa Yankyera | Austin, TX |
| Michael W. Simon MD, PSC | Michael W. Simon, MD | Teresa L. Sutherland  Sandy Hippe  Edgar Guardado-Rosales | Lexington, KY |
| MedPharmics - Platinum - PPDS | Robert Jeanfreau, MD | Shonna James  Kynisha Johnson  Matthew Pitts | Metairie, LA |
| McGill University Health Centre-Vaccine Study Centre | Soren Gantt, MD, PhD, MPH | Mitchell Shiller  Deirdre McCormack | Quebec, Canada |
| Winnipeg Children’s Hospital, HSC-Winnipeg | Sergio Fanella, MD | Lise Bourrier  Barb Fletcher | Manitoba, Canada |
| Children’s Hospital of Eastern Ontario | Charles Hui, MD | Charles Hui, MD  Jason Brophy, MD  Jennifer Bowes | Ontario, Canada |
| Dalhousie University | Scott Halperin, MD | Joanne Langley, MD  Jeannette Comeau, MD | Nova Scotia, Canada |
| ACHIEVE Research Team, Alberta Children’s Hospital, University of Calgary | James D. Kellner, MD | James D. Kellner, MD  Jessica K. Dunn, MD  Joslyn D. Gray, BSc | Alberta, Canada |
| Children's and Women’s Health Centre of British Columbia | Manish Sadarangani, MD | Manish Sadarangani, MD  Hana Mitchell, MD  Helen He, BSc | British Columbia, Canada |
| The Hospital for Sick Children (SickKids) | Sean (Ari) Bitnun, MD | Ari Bitnun, MD  Valerie Waters, MD  Yamna Ali, MD | Ontario, Canada |

Supplementary Methods

Inclusion and Exclusion Criteria

Each participant was required to meet the following criteria for enrollment:

1. Male or female, aged 6 months to <12 years at screening
2. In good general health (based on investigator assessment)
3. Participants who had a chronic disease could be enrolled if the disease was stable (based on investigator assessment) with no change in status nor change in the medications required for disease control in the 6 months prior to screening (based on investigator assessment)
4. Participants’ parent(s) or legally acceptable representative(s) (LAR[s]) understood and agreed to comply with the study procedures and provided written informed consent and were willing to provide assent
5. Height and weight in participants aged <2 years were both ≥ the 3rd percentile according to WHO Child Growth Standards at screening; body mass index in participants aged ≥2 years was ≥ the 3rd percentile according to WHO Child Growth Standards at screening
6. Female participants not of childbearing potential
7. Female participants of childbearing potential could be enrolled in the study if fulfilling all of the following criteria:
8. Had a negative pregnancy test at screening
9. Had practiced adequate contraception or had abstained from all activities that could result in pregnancy for ≥28 days prior to the first dose (Day 1)
10. Had agreed to continue adequate contraception or abstinence through 3 months following the second dose (Day 29) and the third dose (Day 149/booster day 1)
11. Was not currently breastfeeding
12. Participants aged 6 to <12 months were born at full-term (≥37 weeks’ gestation) with a minimum birth weight of 2.5 kg

Participants who met any of the following criteria were excluded from the study:

1. Known history of SARS-CoV-2 infection or known exposure to someone with SARS-CoV-2 infection or COVID-19 within 2 weeks prior to study injection
2. Was acutely ill or febrile 24 hours prior to or at screening. Fever was defined as a body temperature of ≥38.0°C/100.4°F. Participants meeting this criterion could be rescheduled within the relevant study visit window. Afebrile participants with minor illnesses could be enrolled at the discretion of the investigator
3. Prior administration of an investigational or approved CoV (e.g., SARS-CoV-2, SARS-CoV, or Middle East respiratory syndrome coronavirus-CoV) vaccine
4. Undergone treatment with investigational or approved agents for prophylaxis against COVID-19 within 6 months prior to enrollment
5. Known hypersensitivity to a component of mRNA-1273 vaccine or its excipients
6. Had a medical or psychiatric condition that could pose additional risk as a result of participation, or that could interfere with safety assessments or interpretation of results according to the investigator’s judgment
7. History of a diagnosis or condition that could affect assessment of study endpoints or compromise participant safety according to the investigator’s judgment, specifically the following:
8. Congenital or acquired immunodeficiency, excluding (stable) HIV infection as described in inclusion criteria
9. Chronic hepatitis or suspected active hepatitis
10. A bleeding disorder considered a contraindication to intramuscular injection or phlebotomy
11. Dermatologic conditions that could affect local solicited adverse reaction assessments
12. Any prior diagnosis of malignancy (excluding nonmelanoma skin cancer)
13. Febrile seizures (history of a single, simple febrile seizure was allowed in Part 2 of the study for children aged ≥6 years)
14. Received any of the following:
15. Any routine vaccination with inactivated or live vaccine(s) within 14 days prior to first study injection or planned to receive such a vaccine through 14 days following the last study injection. Influenza vaccination was allowed; however, not within 14 days prior to or post–study dose 1 or dose 2. Influenza vaccination was captured within the concomitant medication electronic case report form
16. Systemic immunosuppressants or immune-modifying drugs for >14 days in total within 6 months prior to enrollment (for corticosteroids, ≥1 mg/kg/day or ≥10 mg/day prednisone equivalent if participant weighed >10 kg). Participants could have visits rescheduled for enrollment if they no longer met this criterion within the screening window. Inhaled, nasal, and topical steroids were allowed
17. Intravenous or subcutaneous blood products (red cells, platelets, immunoglobulins) within 3 months prior to enrollment
18. Participated in an interventional clinical study within 28 days prior to screening or planned to do so while participating in the KidCOVE study
19. Was an immediate family member or household member of study personnel, study site staff, or Sponsor personnel

Key Secondary Objectives

Key secondary objectives included geometric mean (GM) values of SARS-CoV-2–specific binding antibodies (bAbs) on Day 1, Day 57 (1 month after Dose 2), Day 209 (6 months after Dose 2), Day 394 (1 year after Dose 2), booster dose (BD) Day 1 (at least 3 months or 6 months after Dose 2), BD Day 29 (1 month after booster dose), BD Day 181 (6 months after booster dose), and BD Day 366 (1 year after booster dose).

Solicited Adverse Reactions

Local adverse reactions (ARs) were pain, erythema, swelling, and axillary swelling or tenderness. Solicited systemic ARs were fever, irritability/crying, loss of appetite and sleepiness in children aged 6-36 months; fever, headache, fatigue, myalgia, arthralgia, nausea/vomiting and chills were assessed for children aged 37 months-11 years. Severity of each AR was graded on a scale of 0 to 4 according to the Toxicity Grading Scale for Healthy Adult and Adolescent Volunteers Enrolled in Preventative Vaccine Clinical Trials [1].

Pseudovirus Neutralizing Assay

Post-vaccination serology samples from participants (KidCOVE) and from a comparator group of young adults (COVE) were tested using a validated reporter virus microneutralization assay (PPD, part of Thermo Fisher Scientific Vaccines Laboratory Services) [2, 3]. This cell-based assay measured SARS-CoV-2 neutralizing antibody inhibition of 293T-ACE2 cell infection by SARS-CoV-2 reporter virus particles (Wuhan-Hu-1 isolate including D614G). Serum antibody concentrations were ascertained by interpolating the mean of the replicate foci forming unit values from the fitted reference standard curve. The final dilution-corrected antibody concentration was the antibody concentration within the quantifiable range of the assay associated with the lowest dilution. Results were reported as final antibody GMC in arbitrary units (AU)/mL [2].

MSD Binding Antibody Assay

MSD assay (SARSCOV2S2P [VAC123]) was used to detect SARS-CoV-2‒specific IgG antibodies that bind to full-length S protein against ancestral SARS-CoV-2 (Wuhan-Hu-1 isolate) and variants including delta (AY.4), and omicron (BA.1) variant using indirect, quantitative, electrochemiluminescence [4].

**Elecsys^®^ Anti-SARS-CoV-2**

Elecsys® Anti-SARS-CoV-2 (Roche) is an immunoassay intended for the qualitative detection of antibodies to SARS-CoV-2 in human serum and plasma and was used to determine SARS-CoV-2 serostatus. The assay uses a recombinant protein representing the nucleocapsid (N) antigen for the determination of antibodies against SARS-CoV-2. Results are reported qualitatively as nonreactive (COI <1.0; negative) and reactive (COI ≥1.0; positive) (https://www.fda.gov/es/media-base/144247/download).

Sample Size

Sample size calculations were performed for antibody response noninferiority tests for children receiving the mRNA-1273 booster dose compared with young adults (18-25 years) receiving the mRNA-1273 primary series. A per-protocol immunogenicity subset sample size of approximately 289 children with pre-booster negative SARS-CoV-2 status (defined as having a negative reverse transcriptase polymerase chain reaction test and a negative serology test [based on bAb specific to SARS-CoV-2 nucleocapsid as measured by Roche Elecsys Anti-SARS-CoV-2 assay] at the time of the booster dose) receiving a booster dose of mRNA-1273 and 289 young adults receiving the mRNA-1273 primary series provided 90% power to demonstrate noninferiority of immune response as measured by antibody GMC at a 2‑sided alpha of 0.05 (assuming an underlying true GMR value of 1.0 and a noninferiority margin of 1.5). The standard deviation of the natural log‑transformed GMC levels was assumed to be 1.5. In addition, SRRs of ≥95% in children receiving mRNA-1273 booster dose and in young adults receiving mRNA-1273 primary series, with a between-group true difference within 4%, provided >90% power to demonstrate noninferiority of immune response by SRR at a 2-sided alpha of 0.05.

Analysis Populations

The safety set included all participants who received an mRNA-1273 booster dose; all safety analyses except for solicited ARs were conducted using the safety set. The solicited safety set consisted of all participants in the safety set who contributed any solicited AR data; the solicited safety set was used for analyses of solicited ARs. The per-protocol immunogenicity subset included participants who received the planned booster dose of mRNA-1273 per schedule, complied with immunogenicity testing schedule, and had no major protocol deviations that impacted key or critical data; the per-protocol immunogenicity subset (consisting of participants who were negative for SARS-CoV-2 before the booster dose) was used for the primary immunogenicity analysis. The comparator population for hypothesis testing comprised a subset of young adults (18-25 years of age) from the per-protocol immunogenicity subset of the COVE study and consisted of participants who were SARS-CoV-2 negative before vaccination.

Seroresponse Rate Analyses

The seroresponse rate (SRR) difference with 95% CI (using the Miettinen-Nurminen score method) between children receiving the mRNA-1273 booster and young adults after the mRNA-1273 primary series was computed, where seroresponse was defined as an increase in titers from below the lower limit of quantification (LLOQ) at baseline to ≥4x LLOQ, or a ≥4-fold increase over baseline in participants with pre-existing baseline titers that were ≥LLOQ. Noninferiority was declared if the lower bound of the 95% CI of the SRR difference between the 2 groups was >–10%. Analyses were performed using SAS software, version 9.4 (SAS Institute).

Supplementary Tables

Table S1. Background information on COVID-19 and the representativeness of study participants

| **Category** | | **Details** |
| --- | --- | --- |
| Disease, problem, or condition under investigation | | COVID-19 in children 6 months-11 years |
| Special considerations related to | |  |
|  | Sex and gender | COVID-19 rates are similar regardless of sex in children aged 6 months-11 years [5]. Epidemiological data on the number of COVID-19 cases by gender identity and in transgender youth are lacking, but the COVID-19 pandemic created particular challenges for the lesbian, gay, bisexual, and transgender (LGBT) community in general [6]. |
|  | Age | Although approximately 15% of the US population is <12 years, about 10% of reported US COVID-19 cases have been observed in children <12 years [5]. Despite extensive efforts to mitigate transmission, COVID-19 hospitalization rates among children <12 years were similar to or slightly lower than those observed for influenza during the 3 influenza seasons observed before the pandemic [7]. Although deaths in children <12 years are much less common than in other age groups, over 1300 COVID-19-related deaths have been observed in children <12 years in the US since the start of the pandemic [5]. This compares unfavorably to the annual US burden of influenza-related deaths in children <12 years (<160 per year) [8]. |
|  | Race or ethnic group | Certain racial and ethnic minority children (eg, black, Hispanic) comprise a disproportionate percentage of hospitalized children with COVID-19 [9-11]. These same groups appear to have increased risk for severe COVID-19 [12]. |
|  | Geography | Globally, over 750 million confirmed COVID-19 cases and nearly 7 million deaths have been reported with substantial regional variation [13]. |
| Other considerations | | Not applicable. |
| Overall representativeness of this study | | The participants included in this study were aged 6 months-11 years. The proportion of male and female participants was similar. Among the 2519 participants aged 6-11 years included in this analysis, 65.8% were white, 11.1% were black, 8.1% were Asian, and 16.9% were Hispanic or Latino. Among the 153 participants aged 6 months to 5 years included in this analysis, 80.4% were white, 2.6% were black, 5.9% were Asian, and 10.5% were Hispanic or Latino. The study was conducted in the US and Canada, but most participants were from sites in the US. |

Table S2. Participants with vaccine-related MAAEs and related severe AEs by age group throughout the entire study period (to June 1, 2023, data cutoff)

|  | **mRNA-1273** | |
| --- | --- | --- |
|  | **Children 6 months to  5 years**  **mRNA-1273 booster 10 µg**  **(n = 153)** | **Children 6 to 11 years**  **mRNA-1273**  **booster 25 µg**  **(n = 2519)** |
| **Vaccine-related MAAEs, n (%)** | 0 | 20 (0.8) |
| COVID-19 |  | 4 (0.2) |
| Injection site cellulitis |  | 1 (<0.1) |
| Serum sickness-like reaction |  | 1 (<0.1) |
| Decreased appetite |  | 1 (<0.1) |
| Headache |  | 3 (0.1) |
| Supraventricular tachycardia |  | 1 (<0.1) |
| Constipation |  | 1 (<0.1) |
| Urticaria |  | 3 (0.1) |
| Alopecia |  | 1 (<0.1) |
| Idiopathic urticaria |  | 1 (<0.1) |
| Arthralgia |  | 2 (<0.1) |
| Myalgia |  | 2 (<0.1) |
| Fatigue |  | 4 (0.2) |
| Pyrexia |  | 3 (0.1) |
| Chills |  | 1 (<0.1) |
| Injection site hematoma |  | 1 (<0.1) |
| Injection site lymphadenopathy |  | 1 (<0.1) |
| Injection site pain |  | 1 (<0.1) |
| Injection site urticaria |  | 1 (<0.1) |
| Non-cardiac chest pain |  | 1 (<0.1) |
| Vaccination site lymphadenopathy |  | 1 (<0.1) |
| Weight decreased |  | 1 (<0.1) |
| **Vaccine-related severe AEs, n (%)** | 0 | 8 (0.3) |
| Headache |  | 3 (0.1) |
| Vomiting |  | 2 (<0.1) |
| Arthralgia |  | 1 (<0.1) |
| Myalgia |  | 3 (0.1) |
| Fatigue |  | 3 (0.1) |
| Injection site pain |  | 1 (<0.1) |
| Pyrexia |  | 2 (<0.1) |
| Chills |  | 2 (<0.1) |

AE, adverse event; MAAE, medically attended adverse event.

Table S3. Participants with AESIs by age group throughout the entire study period (to June 1, 2023, data cutoff)

|  | **mRNA-1273** | |
| --- | --- | --- |
|  | **Children 6 months to 5 years**  **mRNA-1273 booster 10 µg**  **(n = 153)** | **Children 6 to 11 years**  **mRNA-1273**  **booster 25 µg**  **(n = 2519)** |
| **AESIs, n (%)** | 2 (1.3) | 12 (0.5) |
| Epilepsy | 1 (0.7) | 0 |
| Erythema multiforme | 1 (0.7) | 0 |
| Appendicitis | 0 | 2 (<0.1) |
| Ageusia | 0 | 5 (0.2) |
| Anosmia | 0 | 4 (0.2) |
| Febrile convulsion | 0 | 1 (<0.1) |
| Seizure | 0 | 1 (<0.1) |
| Status epilepticus | 0 | 1 (<0.1) |
| Sinus arrhythmia | 0 | 1 (<0.1) |

AESI, adverse event of special interest.

Supplementary Figures

Figure S1. Overview of the study design

**
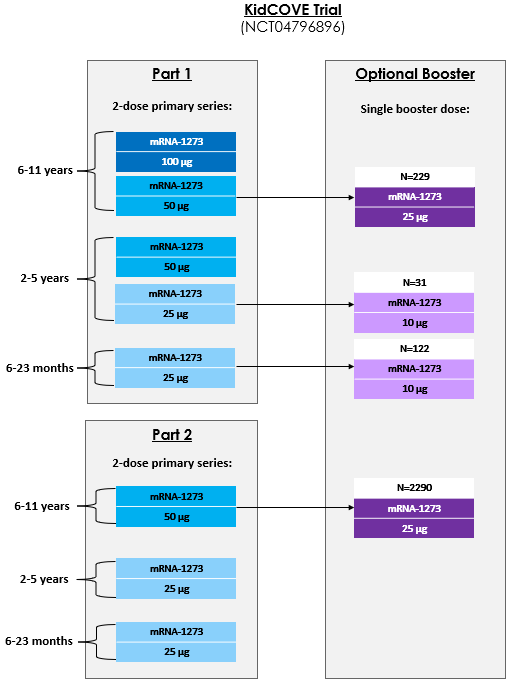
**

Figure S2. Serum neutralizing antibody levels against ancestral SARS-CoV-2 after booster vaccination by SARS-CoV-2 infection status among children 6 months to 5 years (per-protocol immunogenicity set)

Neutralizing antibody responses were assessed pre-booster (baseline) and following booster vaccination (Day 29) among participants with no evidence of current or prior SARS-CoV-2 infection^a^ at the pre-booster visit (n = 76), evidence of current or prior SARS-CoV-2 infection^b^ at the pre-booster visit (n = 20), and overall (n = 103).


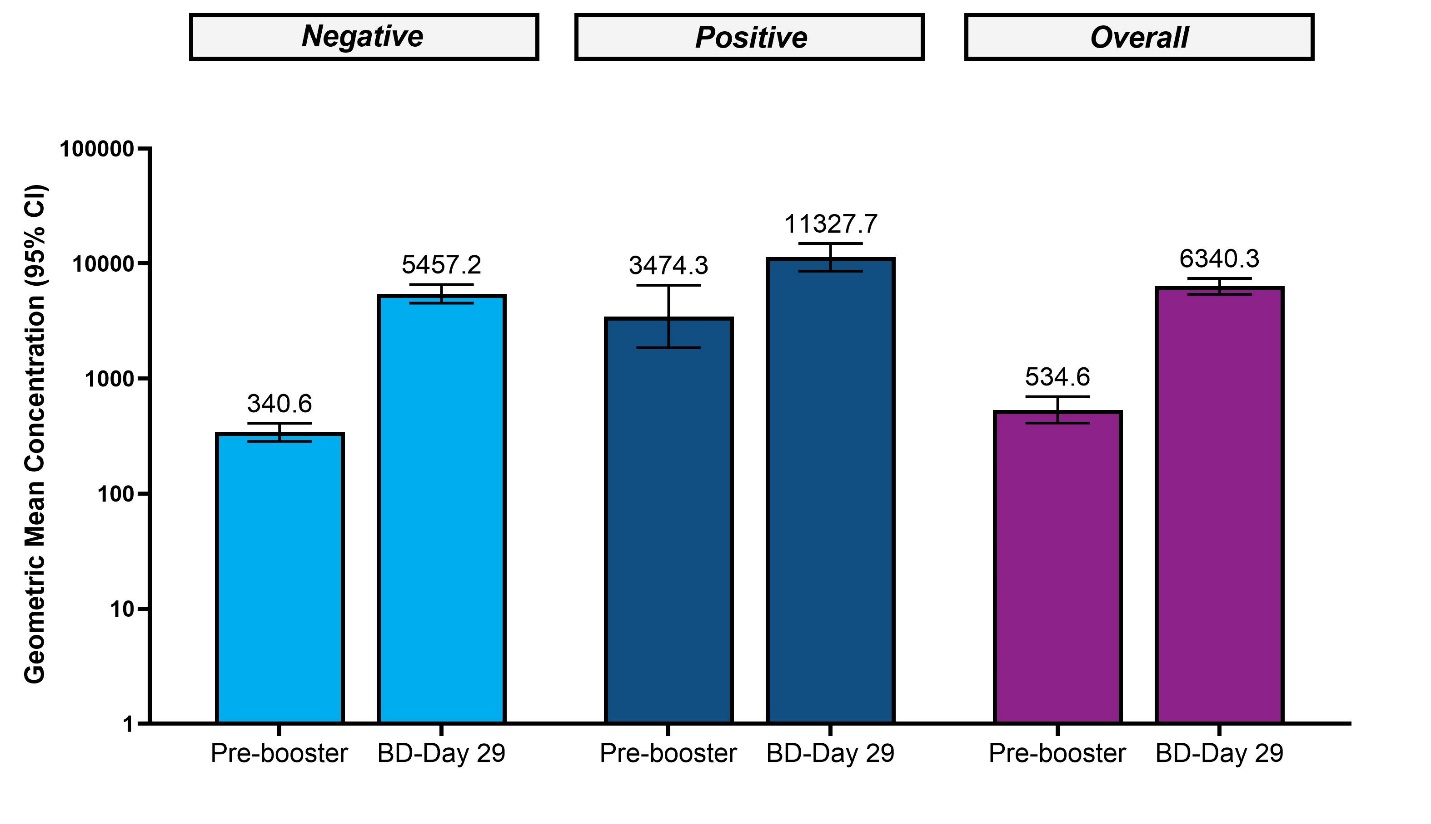


^a^Pre-booster SARS-CoV-2–negative status was defined as having a negative RT-PCR test and negative serology test (based on binding antibody specific to SARS-CoV-2 nucleocapsid as measured by Roche Elecsys Anti-SARS-CoV-2 assay) at the date of the booster dose.

^b^Pre-booster SARS-CoV-2–positive status was defined as having either positive RT-PCR test or positive serology (anti–SARS-CoV-2 nucleocapsid as measured by Roche Elecsys Anti-SARS-CoV-2 assay) on the date of the booster dose.

CI, confidence interval; RT-PCR, reverse transcriptase polymerase chain reaction.

Figure S3. Serum neutralizing antibody levels against ancestral SARS-CoV-2 after booster vaccination by SARS-CoV-2 infection status among children 6-11 years (per-protocol immunogenicity set)

Neutralizing antibody responses were assessed pre-booster (baseline) and following booster vaccination (Day 29) among participants with no evidence of current or prior SARS-CoV-2 infection^a^ at the pre-booster visit (n = 145), evidence of current or prior SARS-CoV-2 infection^b^ at the pre-booster visit (n = 48), and overall (n = 202).


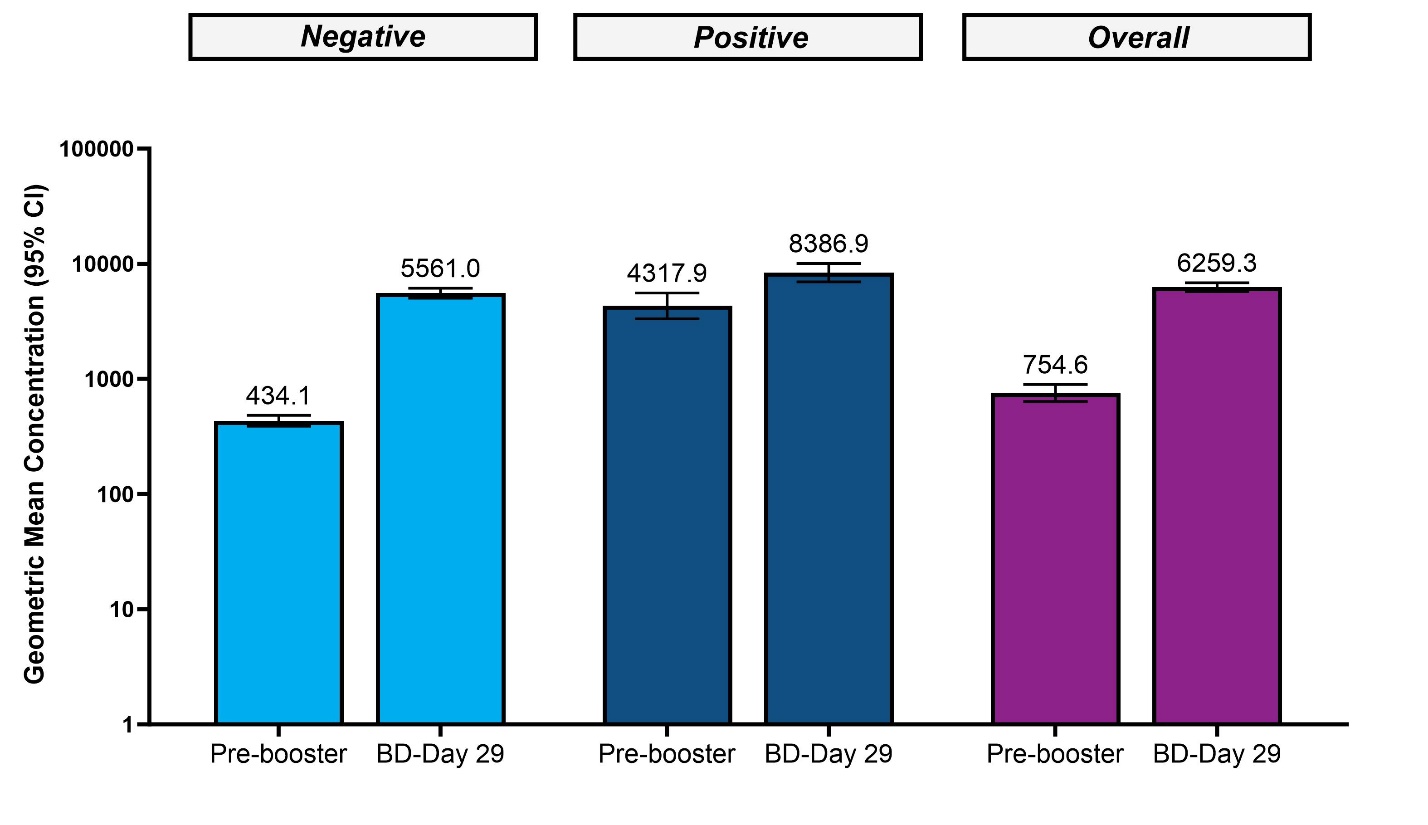
 ^a^Pre-booster SARS-CoV-2–negative status was defined as having a negative RT-PCR test and negative serology test (based on binding antibody specific to SARS-CoV-2 nucleocapsid as measured by Roche Elecsys Anti-SARS-CoV-2 assay) at the date of the booster dose.

^b^Pre-booster SARS-CoV-2–positive status was defined as having either positive RT-PCR test or positive serology (anti–SARS-CoV-2 nucleocapsid as measured by Roche Elecsys Anti-SARS-CoV-2 assay) on the date of the booster dose.
CI, confidence interval; RT-PCR, reverse transcriptase polymerase chain reaction.

Supplementary References

1. U.S. Department of Health and Human Services FaDA, Center for Biologics Evaluation and Research. Toxicity grading scale for healthy adult and adolescent volunteers enrolled in preventive vaccine clinical trials. Available at: <https://www.fda.gov/media/73679/download>.

2. Anderson EJ, Creech CB, Berthaud V, et al. Evaluation of mRNA-1273 vaccine in children 6 months to 5 years of age. N Engl J Med **2022**; 387(18): 1673-87.

3. Bonhomme ME, Bonhomme CJ, Strelow L, et al. Robust validation and performance comparison of immunogenicity assays assessing IgG and neutralizing antibodies to SARS-CoV-2. PLoS One **2022**; 17(2): e0262922.

4. Meso Scale Diagnostics. SULFO-TAG Anti-Human IgG Antibody, 5 Plate. Available at: <https://www.mesoscale.com/en/products/sulfo-tag-anti-human-igg-antibody-5-plate-d21adf/>. Accessed July 17, 2024.

5. Centers for Disease Control and Prevention. COVID data tracker: demographic trends of COVID-19 cases and deaths in the US reported to NVSS. Available at: <https://covid.cdc.gov/covid-data-tracker/#demographics>. Accessed July 17, 2024.

6. Morgan R, Baker P, Griffith DM, et al. Beyond a zero-sum game: how does the impact of COVID-19 vary by gender? Front Sociol **2021**; 6: 650729.

7. Delahoy MJ, Ujamaa D, Taylor CA, et al. Comparison of influenza and coronavirus disease 2019-associated hospitalizations among children younger than 18 years old in the United States: FluSurv-NET (October-April 2017-2021) and COVID-NET (October 2020-September 2021). Clin Infect Dis **2023**; 76(3): e450-e9.

8. Centers for Disease Control and Prevention. FLUVIEW interactive: influenza-associated pediatric mortality. Available at: <https://gis.cdc.gov/grasp/fluview/pedfludeath.html>. Accessed July 17, 2024.

9. Kim L, Whitaker M, O'Halloran A, et al. Hospitalization rates and characteristics of children aged <18 years hospitalized with laboratory-confirmed COVID-19 - COVID-NET, 14 States, March 1-July 25, 2020. MMWR Morb Mortal Wkly Rep **2020**; 69(32): 1081-8.

10. Marks KJ, Whitaker M, Anglin O, et al. Hospitalizations of children and adolescents with laboratory-confirmed COVID-19 - COVID-NET, 14 states, July 2021-January 2022. MMWR Morb Mortal Wkly Rep **2022**; 71(7): 271-8.

11. Shi DS, Whitaker M, Marks KJ, et al. Hospitalizations of children aged 5-11 years with laboratory-confirmed COVID-19 - COVID-NET, 14 States, March 2020-February 2022. MMWR Morb Mortal Wkly Rep **2022**; 71(16): 574-81.

12. Woodruff RC, Campbell AP, Taylor CA, et al. Risk factors for severe COVID-19 in children. Pediatrics **2022**; 149(1).

13. World Health Organization. WHO Coronavirus (COVID-19) dashboard. Available at: <https://covid19.who.int/>. Accessed October 13, 2023.
